# Circular RNA Translation in Cardiovascular Diseases

**DOI:** 10.2174/1389202924666230911121358

**Published:** 2023-10-27

**Authors:** Lijun Wang, Xinxin Cui, Fei Jiang, Yuxue Hu, Wensi Wan, Guoping Li, Yanjuan Lin, Junjie Xiao

**Affiliations:** 1 Institute of Geriatrics (Shanghai University), Affiliated Nantong Hospital of Shanghai University (The Sixth People’s Hospital of Nantong), School of Medicine, Shanghai University, Nantong, 226011, China;; 2 Cardiac Regeneration and Ageing Lab, Institute of Cardiovascular Sciences, Shanghai Engineering Research Center of Organ Repair, School of Life Science, Shanghai University, Shanghai, 200444, China;; 3 Department of Nursing, Union Hospital, Fujian Medical University Union Hospital, Fuzhou, 350001, China;; 4 Fujian Provincial Special Reserve Talents Laboratory, Fujian Medical University Union Hospital, Fuzhou, 350001, China;; 5 Cardiovascular Division of the Massachusetts General Hospital and Harvard Medical School, Boston, MA, 02114, USA

**Keywords:** CircRNAs, cardiovascular diseases, internal ribosome entry sites (IRES), RNA m^6^A, proteins, ribosomal scanning

## Abstract

Circular RNAs (circRNAs) are a class of endogenous functional RNA generated by back-splicing. Recently, circRNAs have been found to have certain coding potential. Proteins/peptides translated from circRNAs play essential roles in various diseases. Here, we briefly summarize the basic knowledge and technologies that are usually applied to study circRNA translation. Then, we focus on the research progress of circRNA translation in cardiovascular diseases and discuss the perspective and future direction of translatable circRNA study in cardiovascular diseases.

## 1 INTRODUCTION

Circular RNAs (circRNAs) are a large class of covalently closed single-stranded RNAs. They are a general consequence of eukaryotic transcription and RNA processing, some of which play essential roles in many cellular events [1-3]. CircRNAs, which were initially considered untranslatable, have also been found to have a particular coding potential [3, 4]. In eukaryotes, cap-dependent translation initiation is the most predominantly mRNA translation initiation mechanism, characterized by recognizing a 5’end m^7^G cap and a 5'-3’ ribosomal scanning step [5]. With the development of algorithms and methodologies (including polysome profiling, mass spectrometry, as well as some molecular/cell biology methods that are applied to circRNA translation identification and verification), circRNA translation to generate peptide or proteins through a cap-independent translation mechanism with internal ribosome entry sites (IRES) or m^6^A RNA modification have been identified [6-8]. The protein/peptide, translated from circRNA, plays important roles in various diseases [9-13]. Here, we briefly introduce the basic knowledge and technologies usually applied to study circRNA translation and then focus on the research progress of circRNA translation in cardiovascular diseases. Finally, perspective on the research progress and future direction of circRNA translation will be discussed.

Ribo-seq is a commonly used translational genome sequencing technology that can detect all translation-competent fragments without other factors [14-16]. This technology obtains the translated sequences encapsulated in ribosomes and then analyzes these RNA fragments [14]. This technology is an important tool for translatome study [16]. To identify a translatable circRNA, polysome profiling separates polysomes by sucrose density gradient centrifugation according to the sedimentation coefficient. Due to the relatively high sedimentation coefficient of the ribosome, RNAs containing different ribosome fragments are well separated. After RNA isolation from polysome fractions, RNA sequencing can be conducted to explore the coding functions of circRNAs [14]. If first enriched circRNA before sequence, potentially translatable circRNA could be identified. For example, using ribo-seq analysis of the human heart, 40 circRNAs that could encode novel proteins are identified [16]. To specifically characterize the precise region of ribosome binding region on circRNA, ribosome footprinting could be used and then followed by RNA-sequencing. To detect a specific candidate circRNA, polysome profiling followed by RT-qPCR in ribosome fractions can also be performed. In addition, the chemical and structural features of circRNA should be characterized by the circular nature of circRNA [2, 17]. Moreover, it is important to identify the encoded peptide across the circRNA’s back splicing site using the mass spectrum or western blot to cross-verify the translation of circRNAs [17].

In addition to identifying circRNA translation from Ribo-seq data and other proteomics analysis methods, some well-established databases that provide circRNA translation information have also emerged (Table **1**). Thus, it is essential to verify the translation of circRNA and select the suitable circRNA for further study from a large crowd of potentially translatable circRNAs. Typically, ORFs have sequences with an initiation codon and stop codon, which enables the ability to encode proteins and polypeptides in eukaryotes [18]. Thus, finding the ORF is always the first step to verifying whether a circRNA is translatable or not. ORFfinder is a tool developed to analyze DNA sequences with ATG start codons and alternative initiation codons. Except for ORFfinder, some tools are available for the prediction and analysis of the coding potential of circRNAs, *e.g*., CPC2 (Coding Potential Calculator), CPAT (Coding Potential Assessment Tool), and PhyloCSF [19-21]. Besides ORF, IRES region or m^6^A methylation sites also needed to be confirmed on circRNA. IRESite, IRESbase, and circAtlas can provide the experimental verified IRES elements, while circBank presents the m^6^A methylation sites on circRNA [22-25].

**Fig. (1):**
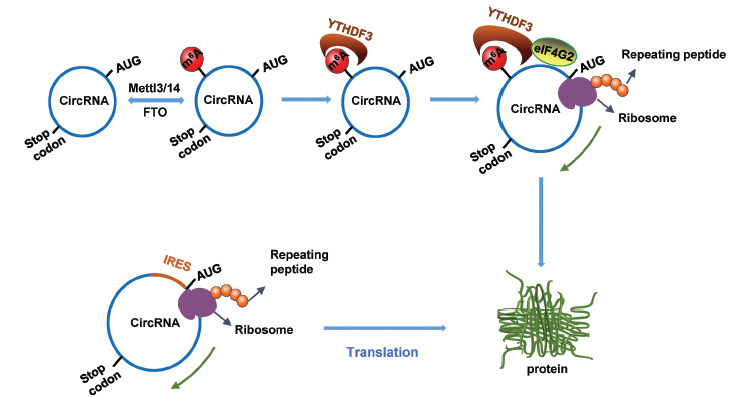
CircRNA translation to peptide/protein through two cap-independent translation mechanisms. Internal ribosome entry sites (IRES) or m^6^A RNA modification on circRNA can recruit translation initiation complex and therefore promote the translation of circRNA into peptide/protein.

Unlike the cap-dependent translation of mRNA translation, circRNAs usually take the cap-independent translation mechanism (Fig. **1**). IRES was originally discovered in viruses and subsequently discovered in eukaryotic cells [26, 27]. IRES are sequences that can skip the cap-dependent structure and directly recruit ribosomes to the inner region of RNA to initiate translation. The IRES-dependent translation mechanism allows encoding proteins and peptides to circRNAs that lack a 5’-cap structure [7]. The first application of IRES in circRNA to encode protein was a synthesized-circRNA which contained an IRES *in vitro* and successfully translated the expected product from the engineered-circRNA, while the circRNA cannot have protein-encoding activities in the absence of an IRES element [28]. With the discovery of circRNAs in recent years, more and more experiments have proved that circRNAs can encode protein/peptide synthesis as mRNA in the cell, among which IRES-dependent translation is one of the important mechanisms that drive circRNA translation [4, 9, 12, 29]. Previously, a split GFP reporter system was developed under the control of the IRES element to determine whether a circRNA can be translatable [7]. circβ-catenin was identified to encode a β-catenin isoform with 370 amino acids in a liver cancer cell in an IRES-dependent manner, and upregulation of circβ-catenin is associated with poor prognosis of liver cancer by preventing β-catenin from GSK3β -mediated ubiquitination and degradation [29]. Similarly, peptides/proteins encoded by IRES-driven circ-SHPRH and circ-FBXW7 have been identified as significant regulators in human diseases [9, 12]. In principle, an IRES-dependent translatable circRNA has several basic characteristics that are indispensable factors, including the existence of an open reading frame followed by IRES, with retention of the start codon and stop codon, as well as the recruitment of translation initiation factors and IRES-transacting factors [7]. Except for the IRES-dependent translation mechanism, m^6^A-dependent translation can also initiate the translation of circRNA. The splinted GFP construct in circular form with an m^6^A-modified “RRACH” sequence can be translated into cells. Modulating the m^6^A methylation in m^6^A-modified circRNA affects the translation efficiency, indicating the involvement of RNA m^6^A modification in the circRNA translation process. RNA m^6^A-driven translation was initiated by the eukaryotic translation initiation factors eIF4G2 and eIF3A, together with the RNA m^6^A binding protein YTHDF3 [6]. Though the importance of cap-independent translation is demonstrated, limited studies have been reported, and further studies are required to discover new translatable circRNA and its physiological significance in the cell.

Cardiovascular disease is one of the leading causes of human death worldwide. Many efforts are devoted to developing new cardioprotective strategies and identifying therapeutic targets [30-32]. In the cardiovascular system, various circRNAs are discovered and found to be involved in the processes of cardiovascular development and diseases [33]. Several studies have investigated the role of translatable circRNA in the heart (Table **2**). CircRNAs circNlgn produced by the neuroligin gene were upregulated in patients with heart disease. circNlgn knockdown significantly reduced human gingival fibroblast proliferation, collagen deposition, and AC16 cardiomyocyte apoptosis. CircNlgn transgenic mice led to heart dysfunction and cardiac fibrosis. Using computational analysis, sucrose gradient isolation, his-Tagged-circNlgn, and western blot confirmed that circNlgn encodes a 173aa polypeptide (Nlgn173). Importantly, Nlgn173, which was translated by circNlgn, can lead to abnormal collagen deposition, cardiac fibroblast proliferation, and reduced cardiomyocyte viability, suggesting that the regulatory role of circNlgn in cardiac remodeling contributed by encoded protein Nlgn173 [13]. An IRES and an ORF encoding a 208aa protein (MyO9A-208) were also found in circ_0036176. MyO9A-208 can mediate the inhibitory effect of circ_0036176 on mouse cardiac fibroblast proliferation. Interestingly, miR-218-5p could inhibit the translation of circ_0036176 into MyO9A-208 at the transcriptional level. The inhibitory effect of circ_0036176 on cardiac fibroblast proliferation was reversed by disrupting MYO9A-208 protein translation, indicating the role of circ_0036176 encoded protein [34]. Circ-ZNF609 was also a translatable circRNA reported to encode proteins and has a critical role in regulating muscle cell proliferation [35]. Inhibition of circ-ZNF609 also attenuated myocardial ischemia-reperfusion injury and doxorubicin-induced cardiotoxicity [36, 37]. It is worth noting that mutations with ATG deletion to disrupt the encoding ability of circ-ZNF609 have a similar regulatory function in modulating cardiomyocyte proliferation and apoptosis, suggesting that circ-ZNF609 itself takes a critical role in cardiomyocytes [37]. Though further studies are required to demonstrate whether circ-ZNF609 encoded protein exerts its regulatory effects on cardiomyocytes in other pathological conditions, this observation reminds us that translatable circRNAs can also exert regulatory effects *via* RNA. Currently, only limited translatable circRNAs have been well-studied in the cardiovascular system. Further investigations are required to demonstrate the underlying physiological and pathological significance of circRNA translation.

## CONCLUSION

In summary, with the advancement of technologies, the diversity of circRNA regulatory mechanisms is gradually revealed. Among these, the translation of circRNA in a cap-independent manner adds a new layer to the protein translation in a eukaryote. At present, our understanding of circRNA translation, either in IRES-dependent or m^6^A-dependent translation, is still in its infancy. Future research focuses on the identification of translatable circRNA as well as its underlying mechanism would contribute to both theoretical understanding and therapeutic approach development in disease management of circRNA translation. Moreover, circRNA-based RNA therapy has the potential to be a powerful therapeutic strategy. Further investigations to take advantage of the cap-independent circRNA translation mechanism design of circRNA-based drugs are of great significance for diagnosing and treating cardiovascular diseases.

## Figures and Tables

**Table 1:** Databases that can be used to identify translatable circRNA.

**Database**	**Applications**	**Internet Site**	**References**
Circcode	A python3-based database of translational circular RNA recognition	https://github.com/PSSUN/CircCode	[38]
TransCirc	Provide an interactive data search engine and visual interface for translatable circRNAs and their translation products	https://www.biosino.org/transcirc/	[39]
riboCirc	Provide comprehensive analysis of circRNA-encoded peptides and visualization of ribosome-associated circRNAs from Ribo-seq datasets	http://ribocirc.com/index.html	[40]
SmProt	A total of 638,958 small proteins were included in the SmProt database, mainly including complete annotations of small proteins encoded by UTRs and non-coding RNAs.	http://bigdata.ibp.ac.cn/SmProt/index.html	[41]
circRNADb	Provide information about the protein-coding potential	http://reprod.njmu.edu.cn/cgi-bin/circrnadb/circRNADb.php	[42]
ORF finder	Search for ORFs in DNA sequence	https://www.ncbi.nlm.nih.gov/orffinder/	-
IRESite	Present information about the experimentally studied IRES elements	http://www.iresite.org/	[22]
IRESbase	Provide information about the experimentally studied IRES elements	http://reprod.njmu.edu.cn/cgi-bin/iresbase/index.php	[23]
circAtlas	circAtlas can provide potential open reading frames and internal ribosomal entry sites	http://circatlas.biols.ac.cn/	[24]
circBank	Provide circRNA protein-coding potential and RNA m^6^A methylation	http://www.circbank.cn/	[25]
circPro	Provide circRNA protein-coding potential *via* high-throughput sequencing data	http://bis.zju.edu.cn/CircPro	[43]

**Table 2:** List of translated circRNAs in the cardiovascular system.

**circRNAs**	**Cap-independent Translation**	**Protein Size**	**Function**	**References**
circNlgn	IRES-driven	173aa	Leads to abnormal collagen deposition, cardiac fibroblast proliferation, and reduced cardiomyocyte viability	[13]
circ_0036176	IRES-driven	208aa	Inhibits cardiac fibroblast proliferation	[34]
circ-ZNF609	IRES/m^6^A	Two isoforms: 250aa or 199aa	Circ-ZNF609 knockdown attenuates myocardial ischemia-reperfusion injury and doxorubicin-induced cardiotoxicity independent of its encoded protein	[36, 37]

## LIST OF ABBREVIATIONS

circRNAs: Circular RNAs
CPAT: Coding Potential Assessment Tool
CPC2: Coding Potential Calculator
IRES: Internal Ribosome Entry Sites
